# Correlation of GLUT4, LEPR , and TNF-a with endometrial receptivity in women with polycystic ovarian syndrome-induced infertility

**DOI:** 10.5937/jomb0-51125

**Published:** 2025-01-24

**Authors:** Dong Yan, Wei Kong, Li Yifei, Liu Yan, Li Lin, Li Yan

**Affiliations:** 1 The Second Hospital Affiliated of Shandong University of Traditional Chinese Medicine, Department of Obstetrics and Gynecology, Ji'nan, China; 2 The Second Hospital Affiliated to Shandong University of Traditional Chinese Medicine, Department of Reproductive Medical Center, Ji'nan, China

**Keywords:** glucose transporter 4, leptin receptor, tumour necrosis factor-a, polycystic ovarian syndrome, infertility, transporter glukoze 4, receptor za leptin, faktor nekroze tumora-a, sindrom policističnih jajnika, neplodnost

## Abstract

**Background:**

To analyze the correlation of glucose transporter 4 (GLUT4), leptin receptor (LEPR), and tumour necrosis factor-a (TNF-α) with endometrial receptivity (ER) in patients with polycystic ovarian syndrome (PCOS)induced infertility to provide clinical evidence for future diagnosis and treatment of PCOS-induced infertility.

**Methods:**

We prospectively enrolled 109 study subjects admitted to The Second Hospital Affiliated of Shandong University of Traditional Chinese Medicine from June 2020 to August 2023, including 42 patients with PCOS-induced infertility (research group), 35 nonpregnant patients with simple PCOS (control group), and 32 nonpregnant healthy women (normal group). GLUT4, LEPR, and TNF-α levels in the peripheral blood were detected in all participants, and their diagnostic value for PCOS in healthy women and PCOS-induced infertility in PCOS patients was analyzed. In addition, the endometrial thickness and endometrial blood flow pulsation index (PI) and resistance index (RI) of patients in the research group were measured. Furthermore, the correlation of GLUT4, LEPR, and TNF-α with ER was discussed.

**Results:**

GLUT4 was lower in the research group compared with the control and normal groups, while LEPR and TNF-α were higher (P<0.05); the control group showed lower GLUT4 and higher LEPR and TNF-α levels than the normal group (P<0.05). The diagnostic sensitivity and specificity of GLUT4, LEPR, and TNF-α combined assay for PCOSinduced infertility in PCOS women were 88.57% and 75.00%, respectively, and those for PCOS in healthy women were 78.57% and 60.00%, respectively (P<0.05). In the research group, GLUT4 was positively correlated with endometrial thickness and negatively linked to RI and PI. At the same time, LEPR and TNF-α were negatively associated with endometrial thickness and positively correlated with RI and PI (P<0.05).

**Conclusions:**

GLUT4, LEPR, and TNF-α are closely related to ER in patients with PCOS-induced infertility, and their combined detection can effectively evaluate the occurrence of PCOS and PCOS-induced infertility.

## Abstract (sr)

**Uvod:**

Cilj je bio analiza korelacije transportera glukoze 4 (GLUT4), receptora za leptin (LEPR) i faktora nekroze tumora-a (TNF-a) sa receptivnošću endometrijuma (ER) kod pacijentkinja sa neplodnošću koju je izazvao sindrom policističnih jajnika (PCOS), kako bi se obezbedili klinički dokazi za buduću dijagnozu i lečenje neplodnosti izazvane PCOS-om.

**Metode:**

Uključili smo 109 ispitanica primljenih u Drugu bolnicu pri Univerzitetu za tradicionalnu kinesku medicinu u Šandongu od juna 2020. do avgusta 2023. godine, uključujući 42 pacijentkinje sa neplodnošću izazvanom PCOS-om (grupa istraživanja), 35 pacijentkinja koje nisu bile trudne sa jednostavnim PCOS-om (kontrolna grupa) i 32 zdrave žene koje nisu bile trudne (normalna grupa). Nivoi GLUT4, LEPR-a i TNF-a u perifernoj krvi su određeni kod svih učesnica, a analizirana je njihova dijagnostička vrednost za PCOS kod zdravih žena i za neplodnost koja je izazvana PCOS-om (kod pacijentkinja sa PCOS-om). Pored toga, izmerene su debljina endometrijuma i pulsacioni indeks (PI) i indeks otpora (RI) krvotoka u endometrijumu kod pacijentkinja iz grupe istraživanja. Takođe je razmotrena i korelacija GLUT4, LEPR-a i TNF-a sa receptivnošću endometrijuma (ER).

**Rezultati:**

Nivoi GLUT4 su bili niži u grupi istraživanja u poređenju sa kontrolnom i normalnom grupom, dok su nivoi LEPR-a i TNF-a bili viši (P<0,05); kontrolna grupa je poređenj'u sa normalnom grupom (P<0,05). Dij'agnostička osetljivost i specifičnost kombinovanog testa za određivanje GLUT4, LEPR-a i TNF-a za neplodnost izazvanu PCOS-om kod žena sa PCOS-om bile su 88,57% i 75,00%, dok su za PCOS kod zdravih žena bile 78,57% i 60,00% (P<0,05). U grupi istraživanja, GLUT4 je bio u pozitivnoj korelaciji sa debjinom endometrij'uma i negativno povezan sa RI i PI. Istovremeno, LEPR i TNF-a su bili negativno povezani sa debljinom endometrijuma i u pozitivnoj korelaciji sa RI i PI (P<0,05).

**Zaključak:**

GLUT4, LEPR i TNF-a su usko povezani sa receptivnošču endometrijuma (ER) kod pacijentkinja sa neplodnošču izazvanom sindromom policističnih j'aj'nika (PCOS), a njihovo kombinovano određivanje može efikasno da proceni prisustvo PCOS-a i neplodnosti izazvane PCOS-om.

## Introduction

Polycystic ovarian syndrome (PCOS) is the most common reproductive endocrine and metabolic disorder in women of childbearing age, which manifests clinically as irregular menstruation (oligomenorrhea or amenorrhea), abnormal ovulation, and polycystic ovarian changes [1]. According to the survey, the global incidence of PCOS among women of childbearing age is as high as 6–10%, showing an increasing trend year by year [2]. Chronic ovulation disorders in PCOS are also important factors causing adverse reproductive outcomes such as repeated pregnancy failures and infertility. Accumulating evidence suggests that poor oocyte quality, repeated implantation failures, and high miscarriage rates all contribute to the difficulty of maintaining a pregnancy in women with PCOS [3]. PCOS-induced infertility is currently a major cause of female infertility, accounting for approximately 30% of all female infertility cases [4]. Endometrial receptivity (ER) refers to the capacity of the endometrium to facilitate embryo adhesion, invasion, and prompt a cascade of adaptive modifications essential for successful implantation. It is widely recognized that alterations in ER preceding embryo implantation indicate pre-existing endometrial irregularities [5]. A significant decline in ER has been observed in patients with PCOS, which is intricately linked to regulatory processes involving oxidative stress, chronic low-grade inflammation, metabolic irregularities, endocrine dysfunctions, and abnormalities in endometrial hormone receptors. Thus, promptly assessing alterations in ER could offer novel insights for diagnosing and treating infertility associated with PCOS.

Glucose transporter 4 (GLUT4), primarily found in adipose and muscle tissue, is regulated by insulin and predominantly located in intracellular storage vesicles in the absence of insulin stimulation [6]. Adequate glucose metabolism is involved in the maturation and differentiation of endometrial cells. GLUT4 acts as a vehicle to transport insulin-mediated glucose, suggesting its potential association with ER in patients with PCOS-induced infertility [7]. Obesity has also been indicated to be one of the important links affecting insulin and glucose secretion [8]. Among them, leptin is a polypeptide hormone secreted by adipocytes, which is positively correlated with fat content and plays a role in hormone secretion, reproduction, inflammation, and other physiological systems and metabolic pathways through binding with its receptors [9]. Tumour necrosis factor-α (TNF-α), a representative substance of inflammatory factors, produces complex biological activities such as promoting cell proliferation and differentiation, apoptosis, immunomodulation, inflammation mediation, anti-tumour action, and cytotoxicity by transmitting information to the nucleus [10]. Although GLUT4, the leptin receptor gene (LEPR), and TNF-α have been confirmed to be closely correlated with PCOS [11] [12] [13], few reports have proposed their relationship with ER in patients with PCOS-induced infertility.

This study undertakes an initial analysis to examine the association between GLUT4, LEPR, and TNF-α levels and endometrial receptivity (ER) in patients experiencing infertility due to polycystic ovary syndrome (PCOS), intending to offer novel insights and recommendations for the future clinical diagnosis and treatment of PCOS-related infertility.

## Materials and methods

### Research participants

We prospectively enrolled 109 study subjects admitted to The Second Hospital Affiliated of Shandong University of Traditional Chinese Medicine from June 2020 to August 2023, including 42 patients with PCOS-induced infertility (research group), 35 nonpregnant patients with simple PCOS(control group), and 32 nonpregnant healthy women (normal group). This study was conducted strictly with the Declaration of Helsinki, and all study subjects signed informed consent forms. Ethical approval from the Ethics Committee of the Second Hospital Affiliated to Shandong University of Traditional Chinese Medicine (NO. 2022021) has been obtained.

### Eligibility and exclusion criteria

Inclusion criteria for the research group: All the included patients (20–40 years old) met the diagnostic criteria for PCOS [14] with normal consciousness, normal sexual activities, no use of any contraceptive measures, and no conception within the past year. In addition, the presence of ovulation abnormalities was confirmed by an ovulation test. Exclusion criteria for the research group: Thyroid hyperprolactinemia and other conditions that cause ovulation disorders; diseasessuch as androgen-secreting tumours, Cushing syndrome, and congenital adrenal hyperplasia that lead to elevated androgen levels; organic diseases of the heart, lung, kidneys, and other vital organs; cardiac and/or renal surgery within 3 months prior to enrollment. Inclusion criteria for the control group: confirmed diagnosis of PCOS, but ovulation test showed normal ovulation; age 20–40 years. The exclusion criteria were the same as those of the research group. The inclusion criteria for the normal group are healthy women with normal ovulatory function and no history of major gynaecological diseases. The exclusion criteria were the same as those of the research group.

### Sample collection and detection

Fasting cubital venous blood (3 mL) was collected from all the subjects at admission, and the bloodserum was obtained by centrifugation after standing at room temperature for 30 minutes. The levels of GLUT4 (Shanghai Kepeirui Biotechnology Co., Ltd.), LEPR (Shanghai Zeye Biotechnology Co., Ltd.), and TNF-α (Shanghai Yiji Industrial Co., Ltd.) were detected by enzyme-linked immunosorbent assay (ELISA) kits. Endometrial thickness, endometrial blood flow pulse index (PI), and resistance index (RI) were measured by colour Doppler ultrasonography.

### Endpoints

The expression of GLUT4, LEPR, and TNF-α in PCOS-induced infertility patients and their correlations, as well as their diagnostic value for PCOSinduced infertility in PCOS patients and for PCOS in healthy women, were investigated. In addition, the correlation of GLUT4, LEPR, and TNF-α with ER in PCOS-induced infertility patients was discussed.

### Statistical methods

Statistical analyses were performed using SPSS24.0 software. The Chi-square test was used forcomparisons of counting data [n(%)], and the independent sample t-test was utilized for comparisons of measurement data (x̄±s). Pearson correlation coefficients analyzed correlations. Diagnostic value was determined using receiver operating characteristic (ROC) curves, and diagnostic effects were evaluated by the area under the curve (AUC; a higher AUC suggests a better diagnostic effect). Logistic binary regression analysis was carried out to obtain the Log(P) formula of GLUT4, LEPR, and TNF-α joint assay, and then ROC curve analysis was carried out. A significance level of P<0.05 was used in all analyses.

## Results

### Comparison of clinical baseline data

The age, body mass index (BMI), family medical history, and other baseline data of the three groups were compared, and no statistical inter-group was identified (P>0.05, Table 1), which confirms the comparability among the three groups.

**Table 1:** Comparison of clinical baseline data.

Group	Age	Body mass index<br>(kg/m^2^)	Duration of disease<br>(years)	Family medical history<br>(with/without)
Normal (n=32)	27.44±3.32	24.01±2.87	–	3 (9.38)/29 (90.63)
Control (n=35)	28.11±4.02	25.41±2.94	3.06±0.76	5 (14.29)/30 (85.71)
Research (n=42)	28.00±2.43	25.55±3.64	2.95±0.66	5 (11.90)/37 (88.10)
F (or t or χ^2^)	0.411	2.400	0.645	0.384
P	0.664	0.096	0.521	0.825

### Comparison of GLUT4, LEPR, and TNF-α levels

**Figure 1:**
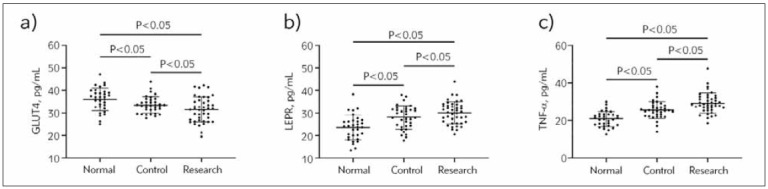
Comparison of GLUT4, LEPR, and TNF-α in healthy pregnant women, PCOS patients, and PCOS-induced infertility patients. a) Comparison of GLUT4, b) Comparison of LEPR, c) Comparison of TNF-α. GLUT4, glucose transporter 4; LEPR, leptin receptor; TNF-α, tumour necrosis factor-α; PCOS, polycystic ovarian syndrome.

After testing, GLUT4 was found to be (31.50±5.57) pg/mL, LEPR was (30.10±4.97) pg/mL, and TNF-α was (29.24±5.35) pg/mL in the research group. It can be seen that GLUT4 was lower, and LEPR and TNF-α were higher in the research group compared to the control and normal groups (P<0.05). Furthermore, compared with healthy controls, patients with simple PCOS had lower GLUT4 and higher LEPR and TNF-α (P<0.05, Figure 1).

### Diagnosis of PCOS-induced infertility by GLUT4, LEPR, and TNF-α

Through Logistic binary regression analysis, the formula

**Figure 2:**
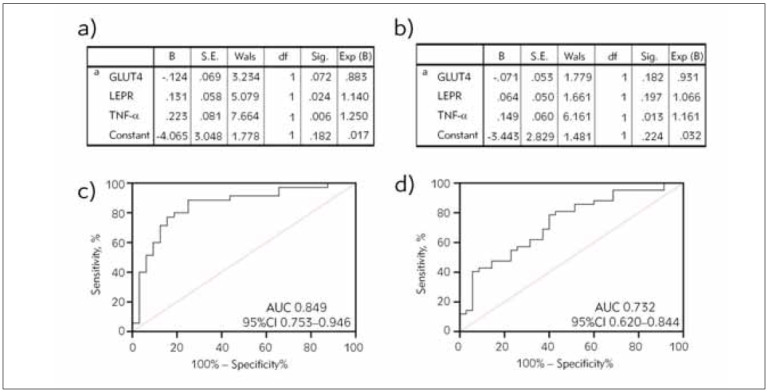
Predictive value of GLUT4, LEPR, and TNF-α for PCOS, PCOS-induced infertility a) Binary logistic regression analysis results of GLUT4, LEPR, and TNF-α combined detection for the occurrence of PCOS-induced infertility in PCOS patients, b) Binary logistic regression analysis results of GLUT4, LEPR, and TNF-α combined detection for PCOS in normal pregnant women, c) ROC curves of GLUT4, LEPR, and TNF-α combined detection for the development of PCOS-induced infertility in PCOS patients, d) ROC curves of GLUT4, LEPR, and TNF-α combined detection for the development of PCOS in normal pregnant women. GLUT4, glucose transporter 4; LEPR, leptin receptor; TNF-α, tumour necrosis factor-a; PCOS, polycystic ovarian syndrome.

Log(P)=-4.065+ (-0.124\timesGLUT4)+ \\ (0.131\timesLEPR)+(0.223\timesTNF-\alpha)

for PCOS-induced infertility by the joint detection of GLUT4, LEPR and TNF-α was obtained (Figure 2a). In healthy women, a joint formula was utilized to evaluate PCOS by combining GLUT4, LEPR, and TNF-α measurements. The formula is: 

Log(P)=-3.443+ (-0.071\timesGLUT4)+ \\ (0.064\timesLEPR)+(0.149\timesTNF-\alpha) (Figure 2b).

The ROC curve analysis showed that the sensitivity and specificity of the formula in diagnosing PCOS-induced infertility were 88.57% and 75.00%, respectively (P<0.05, Figure 2c); for the occurrence of PCOS in healthy women, the joint detection of GLUT4, LEPR, and TNF-α had a diagnostic sensitivity of 78.57% and a specificity of 60.00% (P<0.05, Figure 2d).

### Relationship between GLUT4, LEPR, and TNF-α in patients with PCOS-induced infertility

**Figure 3:**
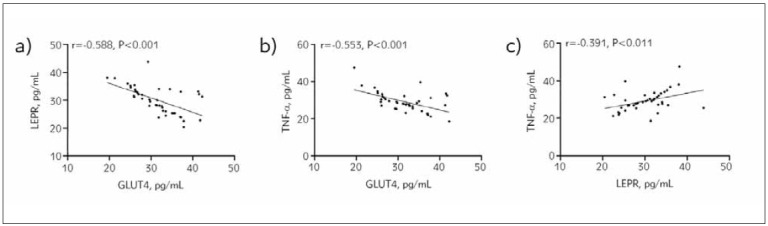
Correlation of GLUT4, LEPR, and TNF-α in patients with PCOS-induced infertility. a) Correlation between GLUT4 and LEPR, b) Correlation between GLUT4 and TNF-α, c) Correlation between LEPR and TNF-α. GLUT4, glucose transporter 4; LEPR, leptin receptor; TNF-α, tumour necrosis factor-α; PCOS, polycystic ovarian syndrome.

According to Pearson correlation coefficients, GLUT4 was negatively correlated with LEPR and TNF-α (P<0.05), while LEPR was positively correlated with TNF-α (P<0.05, Figure 3).

### Correlation of GLUT4, LEPR, and TNF-α with ER in patients with PCOS-induced infertility

**Figure 4:**
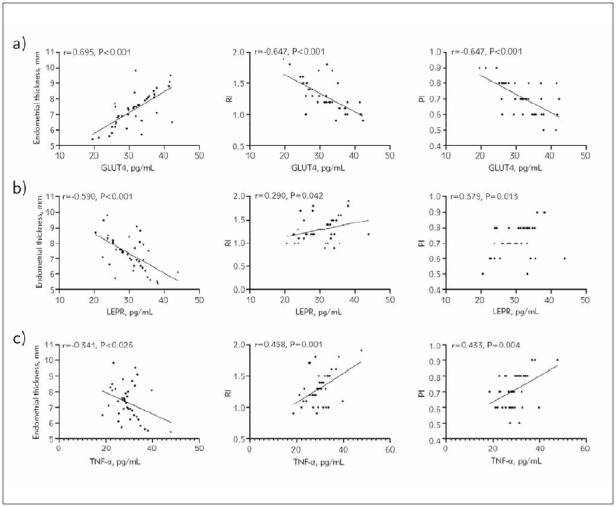
Correlation of GLUT4, LEPR, and TNF-α with ER in patients with PCOS-induced infertility. a) Correlation between GLUT4 and endometrial thickness, RI, and PI, b) Correlation between LEPR and endometrial thickness, RI, and PI, c) Correlation between TNF-α and endometrial thickness, RI, and PI. GLUT4, glucose transporter 4; LEPR, leptin receptor; TNF-α, tumour necrosis factor-α; PCOS, polycystic ovarian syndrome; RI, resistance index; PI, pulsation index; Endometrial receptivity, ER.

Pearson correlation coefficients demonstrated a positive correlation between GLUT4 and endometrial thickness, alongside an inverse relationship between GLUT4 and RI and PI (P<0.05). Conversely, LEPR and TNF-α exhibited negative correlations with endometrial thickness and positive correlations with RI and PI (P<0.05, Figure 4).

## Discussion

PCOS-induced infertility is one of the most important causes of female infertility at present, and effective evaluation of its progression is of great guiding significance for the correct diagnosis and treatment of PCOS-induced infertility in the future [15]. This study identified significant aberrations in the expression levels of GLUT4, LEPR, and TNF-α in individuals experiencing infertility due to PCOS and established their close association with ER in PCOS patients. These findings underscore the importance of these markers for future research and clinical strategies aimed at diagnosing and managing PCOS-related infertility.

First, to confirm the expression of GLUT4, LEPR, and TNF-α in PCOS-induced infertility, we included women with PCOS-induced infertility, those with simple PCOS, and healthy control women as the research subjects. GLUT4 was found to be significantly decreased in PCOS-induced infertility patients, while LEPR and TNF-α were increased, suggesting a strong correlation of GLUT4, LEPR, and TNF-α with the occurrence and development of PCOS-induced infertility. Similarly, patients with simple PCOS showed a decrease in GLUT4 and an increase in LEPR and TNF-α compared with healthy women, which is consistent with the results of previous studies [16] [17] [18] and further supports the potential correlation of GLUT4, LEPR, and TNF-α with PCOS-induced infertility.

GLTU4 transports glucose in muscle and adipocytes, and sufficient glucose metabolism participates in the maturation and differentiation of endometrial cells, suggesting that GLUT4 plays an important role in endometrial gland tissue maturation [19]. We hypothesize that the normal function and metabolism of local endometrium in PCOS patients may be affected, resulting in ER defects, decreased pregnancy rates, and embryo implantation disorders. The decrease in GLUT4 expression reduces its glucose transport ability, weakens the glucose metabolism level of endometrial cells, and triggers the decline of glucose utilization of cells, ultimately affecting endometrial function and causing embryo implantation failure and PCOS-induced infertility [20]. Therefore, the level of GLUT4 in the research group was positively correlated with endometrial thickness and negatively correlated with RI and PI.

Conversely, leptin, an obesity marker released by adipose tissue and a pivotal modulator of insulin sensitivity, has been demonstrated to suppress GLUT4 expression in various tissues and modify glucose uptake by the endometrium, thereby exacerbating PCOS [21]. This study also found an inverse association between GLUT4 and LEPR in patients in the research group, indicating that the lower the GLUT4, the higher the LEPR. Ion channels in the endometrium can mediate endometrial function, among which sodium channels are crucial for ER and embryo implantation [22]. LEPR is reported to down-regulate the expression of endometrial epithelial sodium channels during implantation by activating the STAT3 pathway, and the higher the LEPR, the stronger the inhibition [23]. This is also the reason for the increase in LEPR in the research group. Meanwhile, the higher the leptin level, the worse the ER. Disorders of leptin levels affect ER and embryo implantation, leading to PCOS-induced infertility.

Furthermore, obesity is a well-known metabolic state characterized by chronic inflammation, which is more pronounced in obese patients with high LEPR [24]. This was confirmed by the positive correlation between TNF-α and LEPR in this study. The rise in the levels of inflammatory mediators such as TNF-α can not only reduce ovarian function and normal ovulation but also damage ER in PCOS patients [25]. Oróstica L et al. proposed that pro-inflammatory cytokines can negatively regulate the activation of insulin receptor substrate 1 in the endometrium of PCOS patients by increasing TNF-α, thus interfering with the transmission of insulin signals, which is not conducive to the establishment of normal endometrial function and leads to implantation failures and miscarriages [26]. Furthermore, this finding can corroborate the inverse correlation observed between TNF-α and GLUT4 in this study. Additionally, the connection discovered between TNF-α and ER within the research cohort highlights the link between reduced ER in PCOS-induced infertility and heightened inflammatory responses. The insulin resistance induced by obesity significantly contributes to the dysregulated secretion of pro-inflammatory cytokines, elucidating the interconnected regulation among GLUT4, LEPR, and TNF-α in PCOS-related infertility.

Finally, through ROC curve analysis, we found that the combined detection of GLUT4, LEPR, and TNF-α had an excellent diagnostic effect on the occurrence of PCOS in healthy people and PCOS-induced infertility in PCOS patients, which provides reliable help for more direct, rapid, and accurate clinical evaluation of PCOS and PCOS-induced infertility in the future. Moreover, the high convenience and repeatability of blood sample testing allow for the realization of a wide range of clinical screenings, which is also of great guiding significance for the prevention of PCOS.

Nonetheless, given the restricted number of cases and the reliance on a singular testing approach in this study, the observed outcomes could be incidental. To enhance the robustness of findings, future investigations should encompass a larger sample size, and the levels of GLUT4, LEPR, and TNF-α in individuals with PCOS-induced infertility should be confirmed using alternative detection techniques like polymerase chain reaction. This strategy aims to furnish a more dependable and extensive clinical framework.

## Conclusion

The levels of GLUT4 are reduced in infertility resulting from PCOS, while LEPR and TNF-α exhibit elevated concentrations, all of which strongly correlate with ER in individuals with PCOS-induced infertility. Simultaneously assessing the presence of these three markers can efficiently assess the development of both PCOS and PCOS-associated infertility, furnishing credible clinical evidence. Our findings can be used as a reference to evaluate the progression of PCOS-induced infertility in the future to provide patients with more reliable clinical advice for diagnosis and treatment.

## Dodatak

### Ethical approval

The study protocol was approved by the Ethics Committee of The Second Hospital Affiliated to Shandong University of Traditional Chinese Medicine (No. 2022021).

### Consent to participate

Informed consent was obtained from all individual participants included in the study.

### Consent to publish

All authors gave final approval of the version to be published.

### Availability of data and materials

The data that support the findings of this study are available from the corresponding author upon reasonable request.

### Funding

This study was supported by the Shandong Traditional Chinese Medicine Science and Technology Program (NO.2020M025).

### Author contributions

Yan Li designed the study, Yan Dong and Wei Kong wrote the manuscript, Yifei Li and Yan Liu collected and analyzed data, and Lin Li revised the manuscript. All authors read and approved the final submitted manuscript.

### Conflict of interest statement

All the authors declare that they have no conflict of interest in this work.
